# Principles and applications of optogenetics in developmental biology

**DOI:** 10.1242/dev.175067

**Published:** 2019-10-22

**Authors:** Daniel Krueger, Emiliano Izquierdo, Ranjith Viswanathan, Jonas Hartmann, Cristina Pallares Cartes, Stefano De Renzis

**Affiliations:** 1 European Molecular Biology Laboratory (EMBL), Developmental Biology Unit Meyerhofstrasse 1, 69117 Heidelberg, Germany; 2 Heidelberg University, Faculty of Biosciences, Heidelberg, 69117, Germany

**Keywords:** Embryonic development, Optogenetics, Signaling, Synthetic biology, Tissue morphogenesis

## Abstract

The development of multicellular organisms is controlled by highly dynamic molecular and cellular processes organized in spatially restricted patterns. Recent advances in optogenetics are allowing protein function to be controlled with the precision of a pulse of laser light *in vivo*, providing a powerful new tool to perturb developmental processes at a wide range of spatiotemporal scales. In this Primer, we describe the most commonly used optogenetic tools, their application in developmental biology and in the nascent field of synthetic morphogenesis.

## Introduction

Optogenetics is a technique that, as its names implies, combines genetics and optics to control protein function with light – a principle initially developed by neuroscientists with the aim of controlling neuronal activity with cellular and millisecond-temporal precision (Boyden et al., 2005; Zemelman et al., 2002). It was actually Francis Crick who first suggested that light could help with understanding the complexity of the brain by allowing the activation or inhibition of individual neurons: ‘The ideal signal would be light, probably at an infrared wavelength to allow the light to penetrate far enough. This seems rather farfetched but it is conceivable that molecular biologists could engineer a particular cell type to be sensitive to light in this way’ (Crick, 1999). It took only a few years for the development of the first optogenetic applications for controlling neuronal activity and behavior of a living animal (Banghart et al., 2004; Bi et al., 2006; Boyden et al., 2005; Lima and Miesenböck, 2005; Zemelman et al., 2002).

What Francis Crick did not anticipate, however, is that the technology he had envisioned could also be applied to untangle the complexity of organismal development. Similar to the function of neuronal networks, development of multicellular organisms requires cells to interact in a dynamic manner and to coordinate their behavior through the action of chemical signals. Spatiotemporal regulation is thus a key feature of developmental processes, and optogenetics provides a powerful tool kit for precise subcellular- to tissue-scale perturbations with sub-minute temporal accuracy. By controlling the power and frequency of the light input, optogenetics allows tunable control over protein activity, which can be instrumental to uncover system-level properties that would not be otherwise discoverable using complete loss-of-function perturbations (see Box 1).

In this Primer, we first provide a general description of the most commonly used tools in optogenetics for cell and developmental biology and then discuss how some of these tools have been employed to address developmental biology questions in model organisms.

## Concepts and approaches in optogenetics for cell and developmental biology

**Fig. 1:**
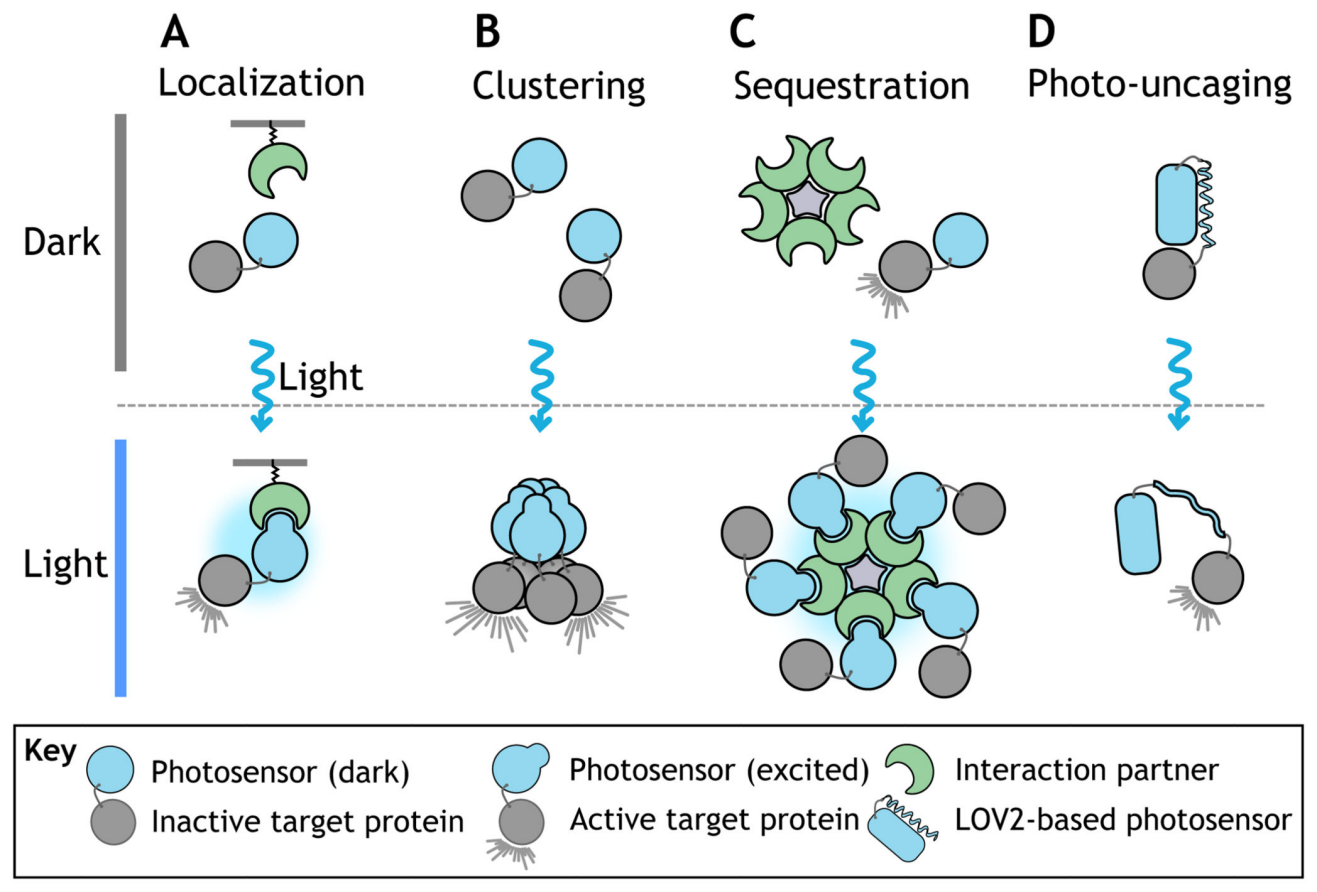
Approaches to controlling protein activity using optogenetics. In all panels, photoreceptors are depicted in blue, their binding partners in green and proteins of interest in gray. (A) Light-induced protein dimerization can be used to recruit a protein of interest to a specific intracellular location, where it can pursue its function. (B) Light-dependent oligomerization (clustering) can induce active functional signaling hubs or inhibit protein function. (C) Light-induced dimerization can also be adopted to sequester a protein of interest away from its site of action. (D) Photo-uncaging based on LOV domains can be used to directly control protein activity with light.

The first optogenetic methods employed rhodopsin-like photosensitive ion channels to stimulate neuronal activity with light (reviewed by Yizhar et al., 2011). Opening of channel pores leads to an influx of ions into the cell, which causes a change in the electric potential across the membrane, and, depending on the channel type, excitation or silencing of neuronal activity. Although optogenetic methods regulating the membrane potential are very useful in neurobiology, their applications in developmental biology are limited as most developmental processes do not rely on changes in membrane potential. The development of a second generation of optogenetic modules based on photoreceptor protein domains that undergo light-induced dimerization/oligomerization or unfolding upon light activation (photo-uncaging) has provided the means to control a wide range of cell and developmental processes (Table 1). The majority of these light-sensitive protein domains derive from plants or cyanobacteria and function in a bio-orthogonal manner when used in animals. When appropriately coupled to a protein of interest, they allow regulation of the protein’s intracellular localization, clustering state, interaction with binding partners, or (in the case of enzymes) catalytic activity, using light of defined wavelengths (Fig. 1). Protein localization is typically controlled using heterodimerization systems consisting of a subcellularly localized anchor that interacts in a light-dependent manner with a cognate photosensitive domain tagged to a protein of interest (Fig. 1A). Relocalization of a target protein to a specific site in the cell can positively regulate its function by enabling it to interact with downstream binding partners and effectors. Alternatively, protein function can be inhibited by sequestering it away from its site of action. The regulation of protein clustering is based on photosensitive protein domains that oligomerize upon activation by light (Fig. 1B). Depending on the target protein, clustering can either positively or negatively regulate protein function, for example by increasing the local concentration of signaling molecules or inhibiting functionality by steric hindrance, respectively. Protein sequestration allows the inactivation of a photo-tagged target protein by capturing it within multimeric protein complexes (Fig. 1C) (Lee et al., 2014). A target protein can also be bioengineered to contain a photosensitive protein domain that unfolds upon light activation causing the exposure of a hidden signaling motif or relieving a protein from allosteric auto-inhibition. This strategy of photo-uncaging allows, for example, direct control over the enzymatic activity of a target protein by light (Fig. 1D). Below, we describe some of the more widely used photoreceptor protein domains and their use in dimerization/clustering and photo-uncaging applications. For a comprehensive overview on available photoreceptors for optogenetic applications, see the OptoBase database (https://www.optobase.org/).

### Light-oxygen-voltage (LOV) domains

**Fig. 2:**
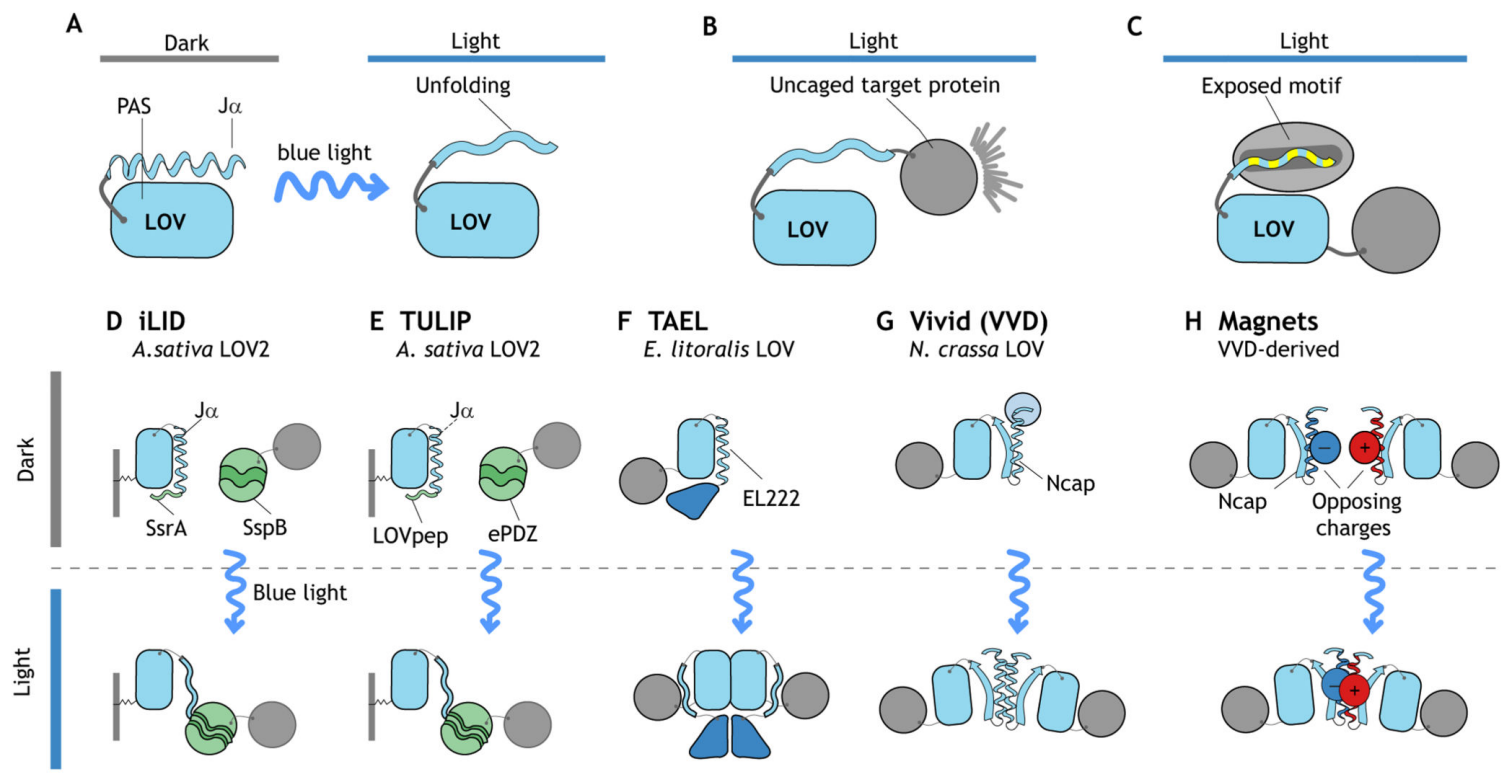
Overview of LOV domain-based optogenetic systems. LOV domains are the most versatile group of photoreceptors. In all panels, LOV domains are depicted in blue, heteromeric interacting partners in green and proteins of interest in gray. (A) The LOV core domain is composed of a Per-Arnt-Sim (PAS) domain that binds to a flavin co-factor and with which it forms a covalent bond upon blue-light illumination. This causes unfolding of an α-helical connector element (e.g. LOV2 Jα). (B) Light-induced conformational changes of LOV domains can be used to photo-uncage a protein of interest and stimulate its activity. (C) The Jα-helix can also be engineered to mask a protein motif that becomes exposed upon light-induced unfolding. (D-H) A variety of different optogenetic dimerization systems are based on LOV domains. These include iLID (D), TULIP (E), TAEL (F), Vivid (VVD) (G) and magnets (H). Whereas iLID and TULIP function by unmasking a protein-interaction domain (e.g. SsrA, LOVpep) upon photoactivation that can be bound by a specific interactor (e.g. SspB, ePDZ) (D,E), TAEL and VVD undergo light-induced homodimerization (through either an adjacent dimerization domain or through the light-responsive N-terminal Ncap fold, respectively) (F,G). (H) Magnets are derived from VVD and engineered to undergo heterodimerization.

LOV domain-containing proteins are a large family found in plants, fungi, algae and bacteria that comprises more than 6000 predicted sequences (Pudasaini et al., 2015) with functional and topological diversity (Glantz et al., 2016). The LOV core domain is composed of a conserved Per-Arnt-Sim (PAS) domain, which is ~110 amino acids long and forms a five-stranded antiparallel β-sheet fold and α-helical connector elements that bind to a flavin chromophore (present in all organisms) as a photoreactive co-factor. Upon blue-light illumination, a covalent bond forms between a cysteine in the PAS domain and flavin (adduct formation), which leads to a conformational change and unfolding of one of the α-helices (e.g. LOV2 Jα) (Crosson and Moffat, 2001; Zayner et al., 2012) (Fig. 2A). This unfolding can be used to expose an engineered recognition motif controlling intracellular protein trafficking, or protein-protein interaction (Fig. 2B,C).

In addition, LOV domains have been engineered to induce protein dimerization (Guntas et al., 2015; Kawano et al., 2015; Strickland et al., 2012; Wang et al., 2012). Different LOV domains possess distinct activation kinetics, sensitivity and relaxation time (reversion to the dark state) (Pudasaini et al., 2015). These parameters should be carefully considered when selecting a LOV domain for optogenetic applications (Table 1). Below, we introduce some of the most commonly used modules for *in vivo* applications, which we also discuss later within the context of development.

#### AsLOV2 and derivatives

AsLOV2 is derived from the LOV2 domain of *Avena sativa* phototropin 1 and has been successfully used for several purposes. For example, in the PA-Rac system, the AsLOV2 domain was used to photo-cage the small GTPase Rac (Wang et al., 2010; Wu et al., 2009). In addition, the LINuS (light-inducible nuclear localization signals)/LANS (light-activated nuclear shuttle) and LEXY (light-inducible nuclear export system) systems were implemented to control nuclear import and export, respectively (Niopek et al., 2014, 2016; Yumerefendi et al., 2015). Finally, protein heterodimerization can be achieved using the TULIP (tunable, light-controlled interacting protein tags) (Strickland et al., 2012) and iLID (improved light-induced dimer) (Guntas et al., 2015) systems (Fig. 2D,E).

#### EL222, VVD and derivatives

EL222 is a naturally occurring transcription factor from *Erythrobacter litoralis* containing a LOV domain that upon blue-light illumination dimerizes and binds to specific regulatory elements of target genes. EL222 was adapted in the TA4-EL222 (TAEL) system to control gene expression in cell culture and during organismal development. The fungal photoreceptor Vivid (VVD) from *Neurospora crassa* and VVD-derived magnets (Kawano et al., 2015; Wang et al., 2012) can be used to control protein homodimerization and heterodimerization, respectively (Fig. 2G,H; Table 1). Similar to EL222, VVD and derivatives were employed to temporarily control gene expression in cell culture systems (Isomura et al., 2017; Kim and Song, 2016; Nihongaki et al., 2017) as well as in the mouse brain (Jung et al., 2019).

### Cryptochrome 2 (CRY2)

CRY2 is another blue-light photoreceptor class (unrelated to LOV domains) from *Arabidopsis thaliana* belonging to the cryptochrome protein family, also present in most animals, which binds flavin adenine dinucleotide as a co-factor (Kennedy et al., 2010). Upon blue-light illumination, CRY2 undergoes photoisomerization and binds to the N-terminal domain of CRYPTOCHROME-INTERACTING BASIC-HELIX-LOOP-HELIX 1 (CIB1), usually referred to as CIBN (Kennedy et al., 2010). By anchoring CIBN to the plasma membrane, or to any other intracellular compartment, it is possible to control the localization of a target protein fused to CRY2 to that location upon photoactivation. The dissociation time of the CRY2-CIBN complex is ~5 min, making this system suitable for most *in vivo* applications (Table 1). When CRY2 is expressed alone (without CIBN), it tends to form homo-oligomers upon photoactivation, often causing inhibition of tagged protein function (Taslimi et al., 2014). CRY2 can also be efficiently activated using two-photon excitation (950 nm), which enables locally restricted illumination patterns with cellular and subcellular precision deep inside tissues in living organisms (Guglielmi et al., 2015; Krueger et al., 2018). Visible blue light (405–488 nm) has the disadvantage that an undefined volume is activated when deeper focal planes are excited, thus limiting the precision with which subcellular optogenetic activation can be achieved (see below).

### Phytochrome B (PHYB)

PHYB belongs to the phytochrome family of photoreceptors found in plants and bacteria, and has the unique feature of being sensitive to red and far-red light. Phytochromes were first identified in *Arabidopsis thaliana* in five different isoforms (PHYA-PHYE) that, upon photoactivation with red light (~650 nm), form heterodimers with phytochrome interacting factors, such as PIF3 and PIF6 (Mathews and Sharrock, 1997; Rockwell et al., 2006). The dissociation of the dimer in the dark is very slow (~20 h), but it can be instantaneously triggered by far-red (~740 nm) illumination, which makes this system ideally suited for applications requiring fast on/off control of protein activity (Table 1). In addition, simultaneous illumination with red and far-red light in partially overlapping spatial patterns allows very precise control of protein activity with subcellular precision, which has been clearly demonstrated in mammalian cell culture (Levskaya et al., 2009) and in developing zebrafish embryos (Buckley et al., 2016). A major limitation of this optogenetic system is the requirement of a chromophore, such as phytochromobilin or phycocyanobilin, which is not present in animal cells and needs to be provided exogenously.

A summary of the optogenetic modules discussed in this Primer and their technical specifications is provided in Table 1. Besides differences in biochemical activity (excitation wavelength, co-factor binding, reversion in the dark, etc.), their molecular size should be also considered, especially when trying to tag small proteins. For example, PHYB is almost four times bigger than GFP, whereas iLID is only half the size (Table 1).

## Optogenetics as a new precision perturbation tool in developmental biology

### Decoding signaling dynamics during development

Spatiotemporal regulation of signaling pathways is key for the generation of diverse responses during the development of multicellular organisms. Owing to limitations in the tools available to manipulate signals at the relevant spatiotemporal scale, it has been so far challenging to study the impact of signal dynamics *in vivo*. What kind of information is encoded in a dynamic signaling system during development? Do cells measure absolute signal levels or changes in concentration over time? Does the frequency of a signaling stimulus matter?

**Fig. 3:**
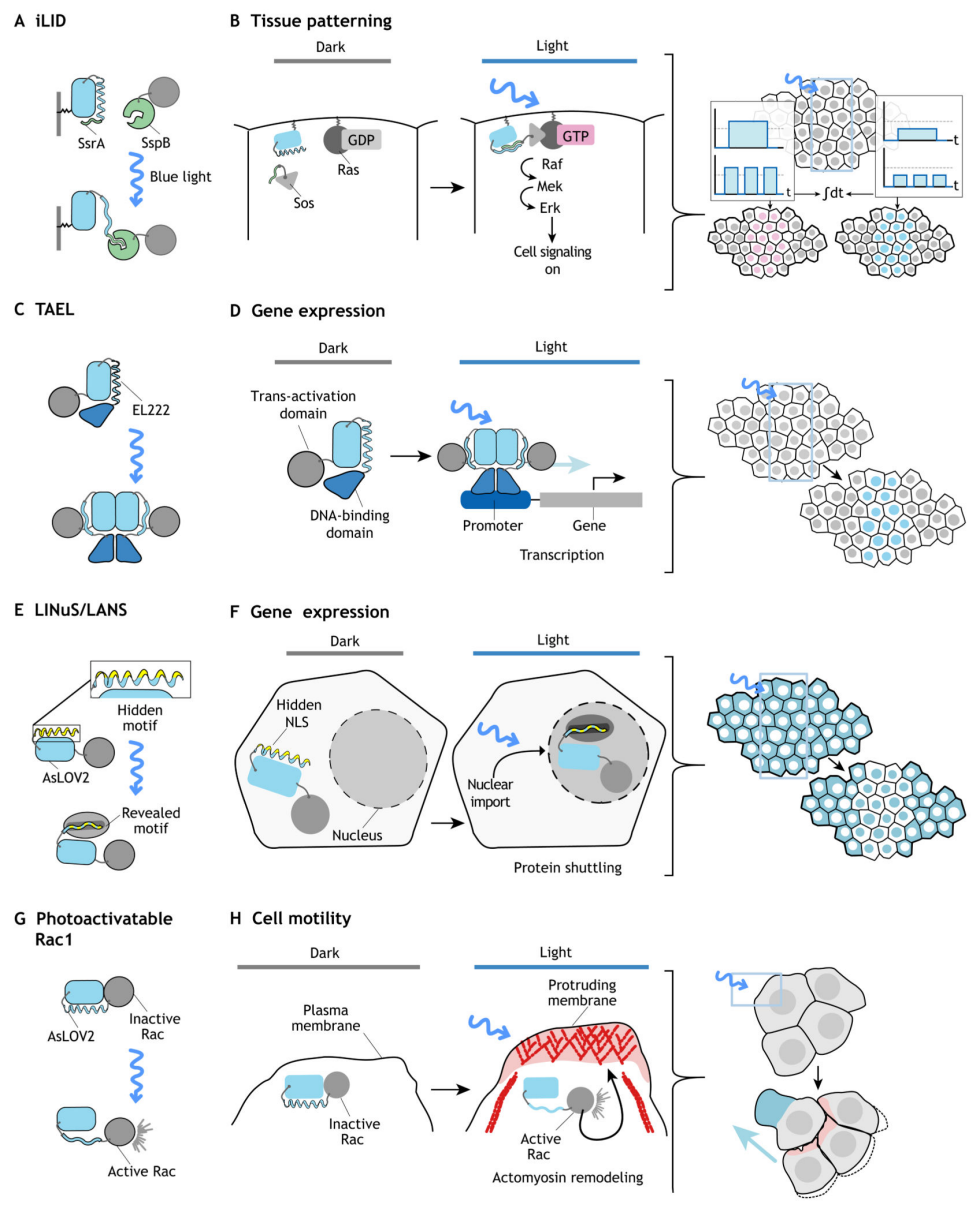
LOV domain-based optogenetic manipulation of animal development. In all panels, LOV domains are depicted in blue, heteromeric interacting partners in green, protein of interests in gray, and an engineered recognition motif in yellow. (A,B) In this example, the iLID heterodimerization system is used to recruit the Ras-GEF Sos to the plasma membrane and activate Erk signaling upon blue-light illumination during early *Drosophila* embryogenesis. By varying the temporal pattern and intensity of light activation, cell signaling and tissue patterning can be controlled. (C,D) The TAEL homodimerization system has been applied in zebrafish embryos to induce gene expression upon light activation. Light-dependent conformational changes in TAEL cause homodimerization and DNA binding of the dimer to a specific promoter region (dark blue) triggering gene expression. (E,F) A Jα of AsLOV2 engineered to contain a nuclear localization signal (NLS) that is exposed only upon light-induced Jα unfolding causes target proteins to shuttle into nuclei. Similarly, the LANS system has been used in *C. elegans* to induce nuclear shuttling of the transcription factor Lin1. (G,H) The small GTPase Rac1 can be photo-caged using the AsLOV2 domain (PA-Rac1). Upon light-induced unfolding of the LOV domain, PA-Rac1 becomes active inducing remodeling of the actomyosin network (red) and lamellipodia formation (pink). PA-Rac1 has been used in *Drosophila* oocytes to guide the movement of border cells using light.

Some of these questions were elegantly addressed by using optogenetic tools to interrogate how cells respond to the concentration and duration of Erk mitogen-activated protein kinase signaling during *Drosophila* embryonic development (Johnson and Toettcher, 2019; Johnson et al., 2017). Erk signaling is required for cells to adopt distinct fates at different positions in the early *Drosophila* embryo. Discriminative upstream factors activate Erk signaling to form head structures at the anterior pole, gut endoderm at the posterior pole, and neurogenic cells at the lateral side. Although genetic approaches allowed a clear demonstration for the requirement of Erk signaling in cell fate specification, they did not allow understanding of how Erk signaling input is differentially conferred to achieve cell fate control. Johnson and co-workers implemented an optogenetic method based on the iLID protein heterodimerization system to precisely regulate Erk signaling in space and time by controlling the activity of the Ras exchange factor Sos, which upon membrane recruitment starts an endogenous signaling cascade that culminates in Erk activation (Fig. 3A,B). Using this method, they demonstrated that cell fate switches in the embryo are triggered by the cumulative dosage of Erk signaling, rather than the duration or amplitude of signaling pulses (Johnson and Toettcher, 2019). These results contradict the ‘transient versus sustained’ model proposed in cell culture, which hypothesizes that cells interpret quantitative differences in signaling dynamics, such as the duration of signaling inputs, as determinants for cell fate specification (Dowdle et al., 2014; Ebisuya et al., 2005; Marshall, 1995; Nakakuki et al., 2010). Instead, *in vivo* the picture emerging from optogenetic control of Erk signaling is that cell fate control is encoded in the total amount of Erk activity integrated over time (Johnson and Toettcher, 2019; Johnson et al., 2017). Similarly, Krishnamurthy et al. implemented the CRY2/CIBN system to photoactivate Raf1, a kinase acting upstream of Erk (Krishnamurthy et al., 2016). Optogenetic activation of Raf1 in *Xenopus* embryos after germ layer specification (a developmental stage during which applications of traditional genetics is technically challenging) induced ectopic tail-like structures in the head region, suggesting that Raf1 activation is sufficient to cause transformation of the embryonic tissue (Krishnamurthy et al., 2016).

During development, signaling pathways (such as Erk) are controlled by morphogen molecules, which are distributed in spatial gradients and provide positional information for tissue patterning and cell differentiation (Wolpert, 1969). The first-identified morphogen, Bicoid (Bcd), patterns cells along the anterior-posterior axis of the early *Drosophila* embryo (Driever et al., 1989). However, the exact time period during which cells integrate positional information has remained elusive. Huang et al. employed optogenetics to temporally control Bcd activity and explore how the dynamics of the Bcd morphogen gradient is interpreted during early development (Huang et al., 2017). By expressing CRY2-tagged Bcd, they rescued *bcd*^−/−^ mutant embryonic development in the dark, indicating that the protein functions normally. When exposed to blue light, however, embryos failed to develop head and thorax structures, resembling the *bcd* mutant phenotype. By restricting Bcd activity to different time windows, they found that targets induced by high Bcd concentration (i.e. targets with low-affinity binding sites) require longer temporal exposure to Bcd, compared with targets induced by low Bcd concentration (i.e. targets with high-affinity binding sites). Thus, optogenetics helped to reveal dynamic aspects of morphogen-sensing mechanisms, namely that cell fates depending on high Bcd concentration also require a longer period of Bcd exposure (Huang et al., 2017).

Comparable optogenetic approaches inhibiting target protein activity in specific tissues were used to demonstrate the temporal requirement of specific factors at distinct stages of development. For example, McDaniel and co-workers show that the pioneer factor Zelda, a master regulator of zygotic genome activation in *Drosophila*, is required throughout the major wave of transcriptional activation and not only to establish competence of *cis*-regulatory regions (McDaniel et al., 2019). In addition, Kaur et al. used CRY2-tagged β-catenin to study the temporal requirement of Wnt signaling in *Drosophila*. They demonstrate that Wnt/β-catenin is required not only for establishing anterior-posterior patterning, but also for the maintenance of this pattern later in development (Kaur et al., 2017).

Viswanathan and co-workers generated a functional CRY2-tagged allele of the Notch ligand Delta (opto-Delta), which allows precise spatiotemporal control over endogenous Notch signaling during *Drosophila* embryonic development (Viswanathan et al., 2019 preprint). They have used this tool to study how Notch signaling input is translated into target gene expression output during tissue differentiation, in particular during mesectoderm (a germ layer giving rise to midline structures of the CNS) induction. Optogenetic activation induces rapid Delta clustering at the plasma membrane and a loss of signaling activity, as a result of *cis*-inhibition of Notch in the receiving cells. By combining precise optogenetic inhibition of Notch signaling and quantitative analysis of nascent nuclear mRNAs of the Notch target gene *sim* (a master regulator of mesectoderm fate) they uncover two distinct relationships between Notch input and *sim* output. At the tissue level, Notch exhibits an analog-like regulatory mode with the level of its activity controlling both the timing and the frequency at which individual nuclei express *sim*. At the level of individual cells, Notch acts in a binary switch-like manner, with a minimum threshold of Notch activity determining whether *sim* is expressed or not. Above a certain threshold, *sim* expression is insensitive to changes in Notch activity. Thus, temporal control over Notch signaling input provided by optogenetics helped to reveal a regulatory mode in which the Notch receptor is a functional integrator of (noisy) analog signals that generates a digital switch-like behavior at the level of target gene expression during tissue differentiation. Viswanthan and co-workers suggest that this may help to minimize spurious target gene expression resulting from transient cell-cell contacts during morphogenetic movements (Viswanathan et al., 2019 preprint).

Optogenetics has been used to spatiotemporally control cell signaling in vertebrates: Sako and co-workers developed a photoactivatable Nodal receptor to study morphogen-signaling regulation in space and time during zebrafish gastrulation. Upon ligand binding, the Nodal receptor dimerizes causing phosphorylation and activation of signal transducers of the SMAD family, which in turn drive Nodal target gene transcription. Sako and co-workers adapted an approach to induce receptor dimerization with light (Grusch et al., 2014) by fusing the intracellular domain of the Nodal receptor (lacking the extracellular Nodal ligand-binding domain) to a LOV domain from *Vaucheria frigida* (Takahashi et al., 2007), which dimerizes upon blue-light stimulation (Sako et al., 2016). Persistent illumination of zebrafish embryos expressing the photoactivatable Nodal receptor triggers endogenous Nodal signaling. Temporal modulation of photoactivation demonstrates that the duration of Nodal signaling is an important determinant for cell fate control during zebrafish gastrulation promoting prechordal plate specification and suppressing endoderm differentiation (Sako et al., 2016).

Together, these studies provide examples of how precise optogenetic manipulation of signaling *in vivo* can reveal novel insights into the dynamics of signaling systems in specific populations of cells during embryonic development.

### Gene expression

The possibility of precisely controlling the location, timing and levels of gene expression could greatly facilitate the study of embryonic development, as it allows the modulation of specific protein abundance at will.

#### TAEL

Reade and co-workers developed a light-gated transcriptional system named ‘TAEL’ that allows the control of exogenous gene expression in zebrafish embryos (Fig. 3C,D) (Reade et al., 2017). The TAEL system is based on a naturally occurring light-responsive transcription factor, EL222, from *Erythrobacter litoralis* that contains a LOV domain and a helix-turn-helix (HTH) DNA-binding domain. In the dark, the LOV domain blocks the HTH domain thereby suppressing DNA binding. Blue-light excitation induces a conformational change in the LOV domain that liberates the HTH domain allowing protein dimerization and binding of TAEL to its cognate DNA regulatory element (C120) resulting in transcription of downstream target genes (Motta-Mena et al., 2014; Reade et al., 2017). TAEL is an effective tool to control gene expression in a variety of different contexts with relatively rapid on/off kinetics, efficacy of which has been demonstrated *in vivo* during zebrafish development. It was used to control the induction of *sox32* expression (a transcription factor controlling endodermal cell fates) in the ectoderm overwriting the endogenous specification program, the expression of the Nodal antagonist *lefty1* allowing time-controlled inhibition of Nodal signaling at different stages of embryonic development, and expression of *cas9* enabling light-induced gene knockout by CRISPR-directed gene editing.

#### Light-inducible transcriptional effectors (LITEs)

Whereas TAEL is designed to activate exogenous genes and functions in a similar way to the GAL4/UAS system, light-inducible transcriptional effectors (LITEs), developed by Konermann and co-workers, allow regulation of endogenous gene expression (Konermann et al., 2013). In this system, CRY2 is tagged to customizable transcription activator-like effector (TALE) DNA-binding domains and CIB1 is fused to the transcriptional activator VP64. Upon blue-light illumination, VP64-CRY2 is recruited to specific genomic regions through TALE-mediated CIB1 interaction (Konermann et al., 2013). This system is effective in regulating both gene expression and chromatin modifications in the brain of living mice using light.

#### LINuS/LANS

Another promising approach to control gene expression is to direct the localization of transcriptional regulators (activators, repressors or epigenetic factors) from the cytoplasm to the nucleus and vice versa. Light-dependent nuclear/cytoplasmic shuttling systems were developed to trigger nuclear translocation in the case of LINuS (Niopek et al., 2014) and LANS (Yumerefendi et al., 2015) (Fig. 3E,F) or to induce nuclear export as in the case of LEXY (Niopek et al., 2016). All these shuttling tools are based on engineered LOV domains that contain cryptic signaling motifs in the LOV domain’s α-helix (e.g. AsLOV2 Jα) that are hidden in the dark and become exposed upon light stimulation allowing interaction with specific regulators of nuclear import/export, which facilitate target protein shuttling. Apart from trafficking signals, motifs such as post-translational modification sequences or degrons, which regulate protein degradation rates, could also be engineered into LOV domains to produce additional tools to control cell and developmental biology processes.

### Collective cell migration

The feasibility of implementing optogenetics to modulate morphogenesis of multicellular organisms was first demonstrated in *Drosophila* and provided a clear demonstration of the power of optogenetics to control single cell behavior *in vivo* (Fig. 3G,H) (Wang et al., 2010). Wang and co-workers investigated the mechanisms controlling collective cell migration by focusing on border cell migration during *Drosophila* oogenesis. Border cells are an interconnected group of six to eight cells that moves a total distance of ~175 μm guided by a complex signaling environment within the *Drosophila* ovary. Wang and co-workers implemented a photoactivatable analog of the small GTPase Rac (PA-Rac1), which was developed to control migration in cell culture (Wu et al., 2009). Rac1 is a pivotal regulator of the actin cytoskeleton controlling cell adhesion, migration and polarity. PA-Rac1 is a fusion protein between Rac1 and the AsLOV2 domain, which in the dark prevents Rac from interacting with its downstream effectors by steric inhibition of the effector-binding site. Light illumination induces a conformational change that liberates Rac1 from this inhibitory state (photo-uncaging) and triggers Rac1 activity. PA-Rac1 provides a tool for the polarized remodeling of the cytoskeleton with full temporal control and subcellular precision. Photoactivation of Rac1 in single cells during border cell migration is sufficient to guide collective cell movements indicating that cells sense direction as a group according to relative levels of Rac activity (Wang et al., 2010). Further studies have also demonstrated the utility of this method to study cell migration in zebrafish embryos (Walters et al., 2010).

During embryonic development, cells receive signaling inputs to gain migratory competence (permissive signaling) and to guide their movements along specific routes (instructive signaling) (Reig et al., 2014). Non-canonical Wnt signaling, for example, is required for coordinated cell migration during metazoan development (De Calisto et al., 2005). To understand better how non-canonical Wnt signaling affects directed cell migration during zebrafish gastrulation, Čapek and co-workers engineered a light-sensitive version of the non-canonical Wnt receptor Frizzled 7 (Fz7) by substituting the intracellular domains of the photoreceptor rhodopsin with the corresponding domains of Fz7 (Čapek et al., 2019). Using this new tool, they demonstrated that uniform photoactivation rescues mesenchymal cell migration during gastrulation of otherwise *Fz7* mutant zebrafish embryos. This result argues that, in addition to its instructive role in controlling cell polarization in epithelial tissues, non-canonical Wnt signaling acts permissively in directing zebrafish mesenchymal migration, without the requirement of localized subcellular activation of Fz7 signaling.

### Tissue morphogenesis

**Fig. 4:**
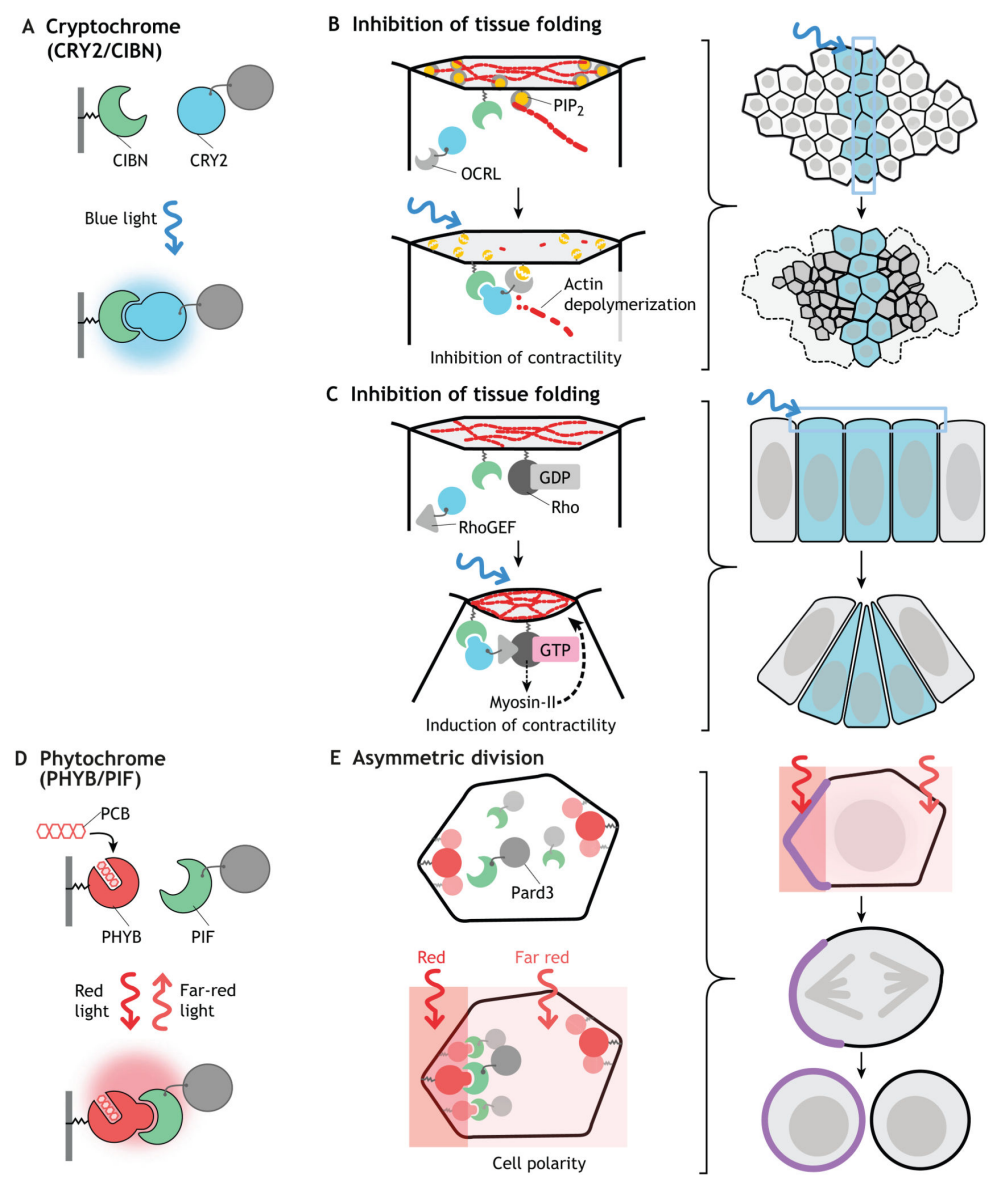
Cryptochrome- and phytochrome-based optogenetic regulation of tissue morphogenesis. In all panels, CRY2 is depicted in blue and PHYB in red, the respective interaction partners in green, and proteins of interest in gray. (A) Upon blue-light illumination, the photosensitive protein CRY2 undergoes a conformational change and binds to its interaction partner CIBN. (B) The cryptochrome system has been applied to recruit the phosphoinositide phosphatase OCRL (tagged with CRY2) to the plasma membrane by triggering the light-dependent interaction of CRY2 with a membrane-anchored CIBN during *Drosophila* gastrulation. At the plasma membrane, OCRL depletes PI(4,5)P_2_ (PIP_2_, yellow circles), which function as anchoring points for cortical actin fibers (red lines). This results in an inhibition of actomyosin contractility and apical constriction during tissue invagination. (C) CIBN/CRY2-mediated recruitment of RhoGEF2 to the apical plasma membrane has been used to trigger Rho signaling upon light exposure culminating in myosin-II-dependent apical constriction and tissue invagination during *Drosophila* embryogenesis. (D,E) The phytochrome system consists of the PHYB photoreceptor, which binds to its interaction partner PIF upon red-light illumination. Far-red illumination causes the PHYB/PIF interaction to dissociate, making the optogenetic system reversible. PHYB activity depends on the plant-specific co-factor PCB. PHYB, anchored at the plasma membrane, recruits the cell polarity determinant Pard3 (fused to PIF) to specific plasma membrane domains (highlighted in purple) upon red light illumination (red region). Illumination of the entire cell with far-red light (pink region) allows PHYB/PIF complex formation only in the region that was simultaneously illuminated with red light. With this strategy it is possible to induce asymmetric inheritance of Pard3 during cell division.

Morphogenesis of tissues requires coordination among cell populations, which leads to the emergence of group properties that are rarely observed in isolated cells, such as symmetry breaking, pattern formation, shape remodeling and regeneration (Xavier da Silveira dos Santos and Liberali, 2019). The extent to which changes in the behavior of single cells influences their neighbors and controls large-scale tissue remodeling has been difficult to study using conventional genetic approaches, owing to limited ability to target individual cells at will. Guglielmi and co-workers set out to examine the dynamics of tissue morphogenesis during *Drosophila* gastrulation, when apical constriction of cells at the ventral midline initiates invagination and formation of the ventral furrow. Apical constriction is induced by contractions of actomyosin filaments anchored at the plasma membrane via actin-binding proteins, localization of which depends on membrane phospholipids and in particular on the phosphatidylinositol phosphate PI(4,5)P_2_ (Bezanilla et al., 2015). Using the CRY2/CIBN system to achieve local control over PI(4,5)P_2_ levels at the plasma membrane, Guglielmi and co-workers showed that local inhibition of apical constriction is sufficient to cause a global arrest of tissue invagination (Guglielmi and De Renzis, 2017; Guglielmi et al., 2015). By varying the spatial pattern of inhibition, they further demonstrated that the coordinated contractile behavior responds to local tissue geometrical constraints (Fig. 4A,B). Together, the results demonstrate that apical constriction is necessary not only to initiate but also to sustain tissue folding and that the geometry of the ventral furrow tissue impacts the way individual cells constrict. These experiments highlight how optogenetics can be used to dissect the interplay between cell-cell interaction, force transmission and tissue geometry during complex morphogenetic processes.

A similar CRY2/CIBN-based system was recently employed by Deneke and co-workers to dissect the connection between cell cycle dynamics and cortical actomyosin contractility during early *Drosophila* embryogenesis. They adopted an optogenetic system to stimulate Rho signaling and apical constriction (Fig. 4C,D; see below) (Izquierdo et al., 2018) to increase the contractility of cortical actomyosin during early syncytial nuclear divisions (Deneke et al., 2019). They used this system to distinguish the role of cortical versus cytoplasmic actin contractility in nuclear positioning, a question that could not be addressed using actin-depolymerizing drugs or conventional genetic approaches that would result in a general impairment of actin dynamics. Using optogenetics they showed that precise spatiotemporal activation of cortical contractility leads to the generation of cytoplasmic flows, which in turn control nuclear positioning and mitotic synchrony. These results argue that cortical and not cytoplasmic contractility drives uniform nuclear positioning and elucidate a self-organized mechanism that links cell cycle oscillators and embryo mechanics.

## Subcellular optogenetics

The spatial resolution of optogenetics is limited by the resolution and precision of the applied optical illumination device as well as the diffusion of the optogenetic components. In simple 2D systems, such as cultured cells, subcellular photoactivation can be achieved by optimizing the light power, frequency and duration of illumination (Benedetti et al., 2018; Meshik et al., 2019). However, many research questions in developmental biology require spatially confined perturbations with subcellular precision within the depth of a tissue with a complex shape. Below, we discuss strategies to achieve subcellular optogenetic control in deep focal volumes and in tissues with curved morphology.

**Fig. 5:**
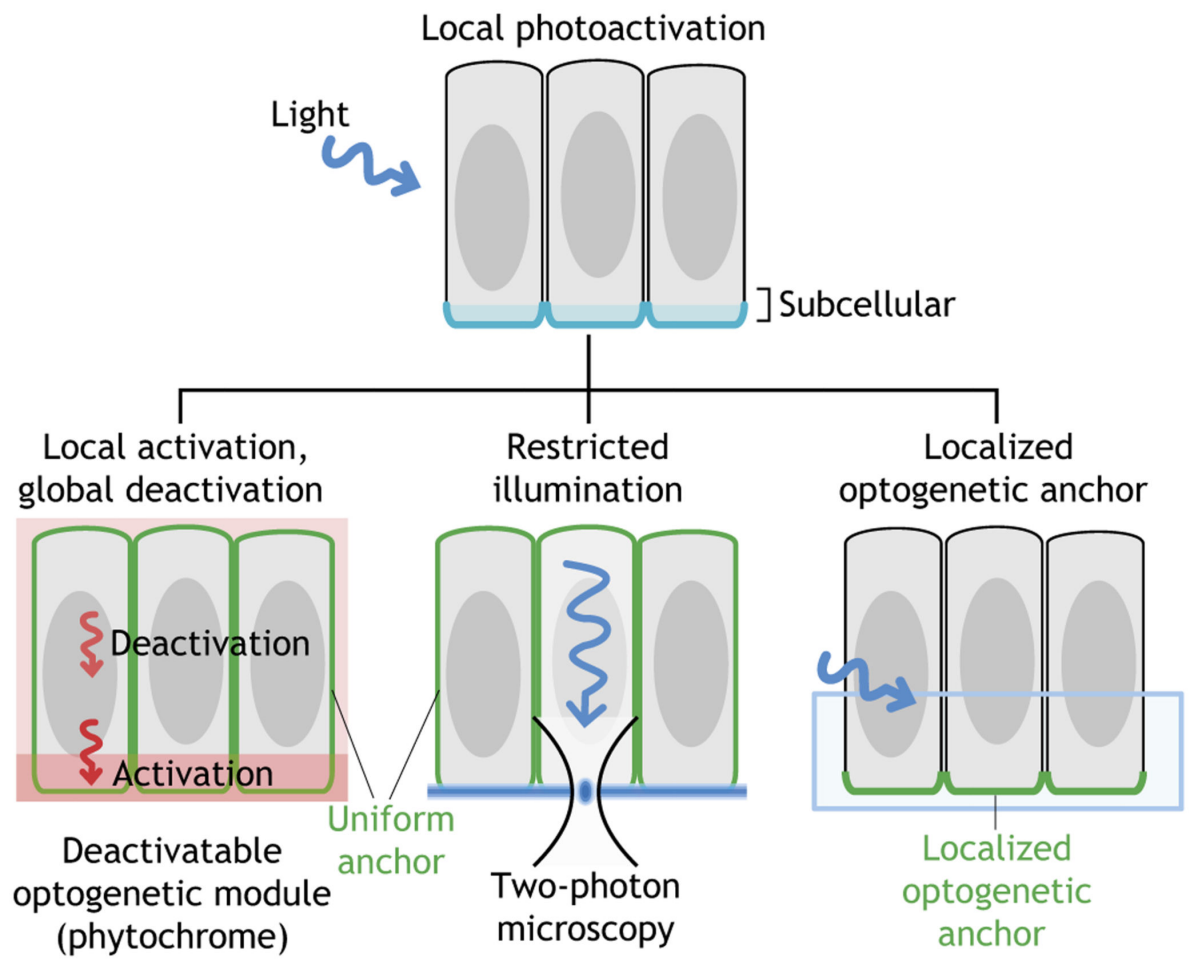
Subcellular optogenetics. Three different approaches have been so far employed to achieve subcellular photoactivation (activated photoreceptor colored in blue, membrane-anchored components in green). Left: Using the PHYB photoreceptor anchored uniformly at the plasma membrane, it is possible to locally photoactivate a subcellular region. Red light-induced PHYB/PIF6 dimer formation is approximately seven times faster than far-red light-induced dissociation. Stimulation of a subregion of interest using red light (red region) with simultaneous deactivation of the whole cell using far-red light (pink region) results in locally restricted photoactivation. Middle: Two-photon excitation using near-infrared light enables locally restricted light delivery (blue blurred line) and photoactivation deep inside living tissues by temporally and spatially restricting the laser light to a focal volume in the femtoliter range. Right: Subcellular optogenetic activation can also be achieved in tissues of complex morphology by engineering an optogenetic anchor in such a way that it localizes only to the site of the cell where optogenetic activation is desired (green cell outline). Components of the cell polarity machinery are ideal candidates for designing optogenetic anchors, as recently demonstrated by the use of PatJ to manipulate myosin-II activity specific at the cell base during *Drosophila* gastrulation. Using this approach, even whole-cell photoactivation results in a locally confined activation of the optogenetic system. See text for more details.

Taking advantage of the reversible properties of the phytochrome system, Buckley and co-workers successfully demonstrated the ability to control cell polarity by rapidly and reversibly recruiting polarity proteins to specific subcellular regions in the depth of a living zebrafish embryos (Fig. 4D,E and Fig. 5). The establishment and maintenance of epithelial apicobasal polarity is a tightly regulated process, which is of key importance during organismal development. Its misregulation causes loss of epithelial integrity, increased cell motility and neoplastic transformation. In their experiments, Buckley and co-workers controlled the localization of the apical polarity protein Pard3 with subcellular precision in the embryo’s enveloping layer epithelium during neural tube formation (Buckley et al., 2016). To achieve this, PHYB was anchored at the plasma membrane through a CAAX (prenylation) anchor and Pard3 was tagged with PIF6. Localized illumination in a region of the plasma membrane using red light of 650 nm caused recruitment of Pard3 to that location, whereas simultaneous global illumination in with far-red light at 750 nm caused dissociation of the complex elsewhere. As red light-induced PHYB/PIF6 dimer formation is approximately seven times faster than far-red light-induced dimer dissociation, Pard3 can be recruited to subregions of the plasma membrane. Importantly, this method allows the manipulation of cell polarity at will *in vivo*, which could be instrumental for future research studying the interplay between cell polarity and tissue morphogenesis. However, as already mentioned above, the phytochrome system requires the addition of a chromophore, which can pose some technical challenges, especially in organisms such as the *Drosophila* embryo that are not permeable to exogenously applied molecules.

To overcome such limitations, Krueger and co-workers developed an approach based on the use of a subcellular-localized CIBN anchor and two-photon illumination, which allows localized photoactivation patterns in tissues with complex morphology, such as folded epithelia in the gastrulating embryo (Fig. 5). Most studies investigating ventral furrow formation during *Drosophila* gastrulation focused on apical constriction and the upregulation of the molecular motor myosin-II at the apical surface, but it was unclear whether additional regulation of myosin-II at the basal surface is also required. Computer simulations predicted a requirement of basal relaxation for completing tissue invagination (Polyakov et al., 2014); however, owing to the lack of genetic mutations interfering specifically with the basal pool of myosin-II, it was impossible to test these models experimentally. Kruger and co-workers used their subcellular optogenetic system to precisely manipulate Rho signaling and myosin-II activity at the basal surface of the invaginating cells in *Drosophila* (Krueger et al., 2018). Indeed, they could specifically counteract the loss of basal myosin-II during ventral furrow invagination and demonstrate that maintaining myosin-II levels at the basal surface inhibits apical constriction, cell shape changes and tissue invagination (Krueger et al., 2018). Importantly, their method not only allows for spatial precision, but also permits quantitative control of myosin-II levels.

## Using optogenetics to reconstruct morphogenesis

**Fig. 6:**
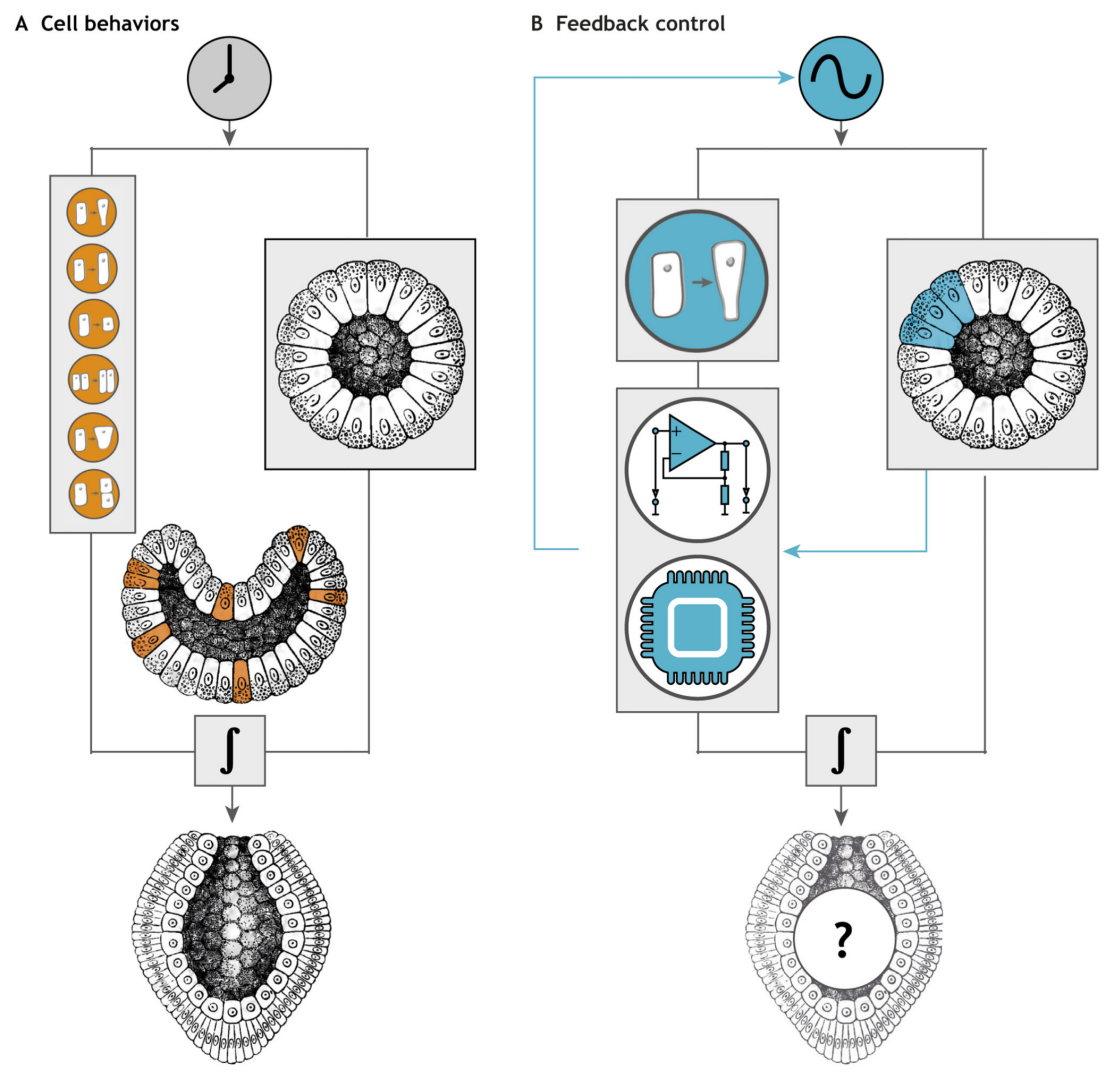
Reconstructing morphogenesis using synthetic biology approaches. (A) Morphogenesis relies on a common set of mechanisms (modules) involving changes in cell behaviors that occur at specific time points and locations, and that give rise to highly complex forms and patterns. (B) By enabling the delivery of precise spatiotemporally controlled inputs, optogenetics allow individual modules to be triggered at will and determine the minimum set of requirements sufficient to drive morphological remodeling. Computerized feedback control could be used to automatically tune optogenetic inputs in real time according to the desired morphogenetic outputs (synthetic morphogenesis). Such an experimental set-up has been recently developed to achieve robust perfect adaptation (RPA) (Aoki et al., 2019) of gene expression in single *Saccharomyces cerevisiae* yeast cells (Rullan et al., 2018). This combination of optogenetic and control theory concepts should allow us to eventually reconstruct complex morphogenetic processes and build synthetic embryos. The embryos depicted in this figure represent the corral *Monoxenia darwinii* during gastrulation as drawn by Ernst Haeckel (Haeckel, 1891).

The ability to manipulate signaling systems and cell behavior with spatiotemporal precision provides the potential to study organismal development not only by interfering with the normal series of events driving morphogenesis (e.g. defining the necessary conditions), but also to guide it and reconstruct it (e.g. defining the sufficiency conditions). The modular nature of morphogenesis implies that it should be possible to single out individual modules, determine the minimum set of requirements that are sufficient to drive morphological remodeling, and eventually reconstruct morphogenesis (synthetic morphogenesis; Fig. 6). Recently, Izquierdo and co-workers used optogenetics to reconstitute tissue invagination during *Drosophila* embryonic development in tissues that otherwise would not undergo internalization (Izquierdo et al., 2018). Using two-photon stimulation of RhoGEF2 tagged with CRY2 and a CIBN plasma membrane anchor, Rho signaling can be triggered to activate myosin-II and thus local cell contractility (Fig. 4C,D). Precise spatial and temporal activation of Rho signaling at the apical surface of epithelial cells on the dorsal side of the embryo is sufficient to trigger apical constriction and tissue folding independently of any pre-determined condition or differentiation program associated with endogenous invagination processes. The resulting optogenetics-guided furrows can be triggered at any position along the dorsal-ventral or anterior-posterior embryo axes in response to the spatial pattern and level of optogenetic activation. In addition, rectangular patterns of photoactivation cause cells to constrict anisotropically, whereas squared patterns cause isotropic constriction, which demonstrates the impact of tissue geometry on individual cell behavior. By tuning the strength of Rho signaling activation, different contractile behaviors can be induced: discontinuous optogenetic activation results in pulsatile apical constrictions, whereas sustained activation induces continuous apical constriction and invagination (Izquierdo et al., 2018). These results demonstrate how optogenetics can be used to reconstruct morphogenesis and study input–output relationships, by coupling signaling systems and tissue shape changes during embryonic development.

## Concluding remarks

In this Primer, we have illustrated how optogenetic techniques can be used in model organisms to address developmental biology questions, emphasizing the unique advantages of precise spatiotemporal perturbations. As with any other technique, optogenetics is not free of limitations. In particular, expression levels and dark-state activity of new optogenetic probes (i.e. the extent to which an optogenetic module is active prior to photoactivation) need to be carefully assessed. When considering stimulation of signaling pathways, it is advisable to combine such perturbations with corresponding downstream biosensors to ensure that pathway activity is within the physiological range. Additionally, the diffusion of photoactivated optogenetic modules can cause complication especially for studying extracellular morphogen signaling or long-range transport within cells. This could be overcome by using the phytochrome system, which allows activation in the desired region and simultaneous deactivation elsewhere. Although optogenetics is only a technique, the results discussed in this article do suggest some common new themes emerging from the use of this methodology to study the complex question of how multicellular organisms develop. Optogenetics allows us to establish very direct cause-effect relationships between gene activities and developmental phenotypes and to decode the engineering principles controlling cell fate decisions and morphogenesis. The possibility of modulating signaling pathways at will with cellular precision *in vivo* means that we have now the ability to reverse engineer and guide organismal development to the extent that we should be able in the near future to build synthetic embryos (Fig. 6). This will allow us to both test theories of morphogenesis and also facilitate the design of tissues with potential applications in regenerative medicine.

## Figures and Tables

**Table 1:** Physico-chemical properties of the most commonly used optogenetic modules in developmental biology

Module	Component(s)	Excitation peak	Reversibility	Reversion in dark	Co-factor	Size (kDa)	Molecular function	Advantages	Disadvantages	Selected *in vivo* application(s)
Cryptochrome (Kennedy et al., 2010)	CRY2/CIBN	450 nm	Stochastic	~5 min	FAD	CRY2: 57 kDa; CIBN: 20 kDa	Heterodimerization; clustering	Easy to implement; CRY2 alone can form oligomeric clusters	Incompatible with GFP*; large tag size	Cell contractility, *Drosophila* (Deneke and Di Talia, 2018; Guglielmi et al., 2015; Izquierdo et al., 2018; Krueger et al., 2018, 2019)
										Differentiation, *Drosophila* (Huang et al., 2017; McDaniel et al., 2019); cell signaling, *Drosophila* (Kaur et al., 2017)
										Cell signaling, *Xenopus* (Krishnamurthy et al., 2016).

Phytochrome (Levskaya et al., 2009)	PHYB/PIF6	660 nm	Light-induced: 750 nm	~20 h	Phytochromobilin/phycocyanobilin (exogenous)	PHYB: ~100 kDa; PIF6: 11.5 kDa	Heterodimerization	Can be specifically switched off with light (700 nm); compatible with GFP fluorescent reporters	Needs an exogenous co-factor; requires optimization for implementation (protein levels, etc.); large tag size	Cell polarity, zebrafish (Buckley et al., 2016)

iLID (Guntas et al., 2015)	AsLOV2/SspB	450 nm	Stochastic	Tunable	FMN	AsLOV2: 16 kDa; SspB: 13 kDa	Heterodimerization	Tunable kinetics; small tag size; easy to implement	Incompatible with GFP*	Cell signaling, *Drosophila* (Johnson and Toettcher, 2019; Johnson et al., 2017)

TULIP (Strickland et al., 2012)	AsLOV2/ePDZ	450 nm	Stochastic	Tunable	FMN	AsLOV2: 16 kDa; ePDZ: 21 kDa	Heterodimerization	Tunable kinetics; easy to implement	Incompatible with GFP*	Cell division, C. elegans (Fielmich et al., 2018)
										Organelle trafficking, *C. elegans* (Harterink et al., 2016)
										Differentiation, sea urchin (Uchida and Yajima, 2018)

Vivid (VVD) (Wang et al., 2012)	VVD	450 nm	Stochastic	Tunable	FAD	20 kDa	Homodimerization	Homodimer formation; tunable kinetics	Incompatible with GFP*	Cell signaling, cell culture (Isomura et al., 2017)

Magnets (Kawano et al., 2015)	pMag/nMag	450 nm	Stochastic	Tunable	FAD	16 kDa	Heterodimerization	Wide range of tunable kinetics (seconds to hours)	Incompatible with GFP*	Gene expression, mouse (Jung et al., 2019)

TAEL (Motta-Mena et al., 2014)	EL222	450 nm	Stochastic	~1 min	FMN	23 kDa	Exogenous gene expression	Homodimer formation; optimized for exogenous gene expression in zebrafish	Incompatible with GFP*	Gene expression, zebrafish (Reade et al., 2017)

LITE (Konermann et al., 2013)	TALE-CRY2/CIB1-VP64	450 nm	Stochastic	~5 min	FAD	TALE-CRY2: 162 kDa; CIB1-VP64: 50 kDa	Endogenous gene expression	Optimized for modulation of endogenous gene expression	Incompatible with GFP*	Gene expression, mouse (Konermann et al., 2013)

LINuS/LANS (Niopek et al., 2014; Yumerefendi et al., 2015)	AsLOV2	450 nm	Stochastic	~5 min	FMN	18 kDa	Protein shuttling	Enables light-induced nuclear import	Needs optimization^‡^; incompatible with GFP*	Gene expression, *C. elegans* (Yumerefendi et al., 2015)

The photosensitive component of the respective optogenetic module is underlined in the ‘Component(s)’ category. FAD, flavin adenine dinucleotide; FMN, flavin mononucleotide.

* Owing to spectral overlap.

‡ Optimization requires addition or removal of endogenous shuttling signals, such as NES or NLS.

## Box 1 Probing complex systems with optogenetics

Developing systems are governed by complex molecular and cellular interaction networks laden with non-linearity and feedback. Even relatively simple interaction networks featuring feedback and non-linearity may exhibit a diverse range of complex behaviors (Isomura et al., 2017). For example, the motif shown in A with dependencies shown in B can produce both equilibrium (C, left) and oscillatory (C, right) behaviors depending on parameters. In such systems, a complete knockout of a single component may propagate in a domino-like fashion to cause a total breakdown of the entire system, a sort of cascade failure by analogy to artificial networks (Buldyrev et al., 2010). The outcomes of such drastic events provide only limited information about the dynamic functioning of the unperturbed system (D). Optogenetics serves to overcome this issue in two ways. First, it facilitates time-resolved experiments to measure the immediate impact of a sudden perturbation (E) rather than the long-term consequences of a permanently missing component (D). Second, it allows finely tuned low-magnitude perturbations that may not trigger total system breakdown and ‘cascade failures’ (F). Both approaches have the potential to provide crucial insight into the system’s wiring and the resulting complex dynamics. Precise optogenetic perturbations thus offer a complementary approach to traditional genetic and chemical perturbations allowing the study of developing systems in a holistic, rather than a reductionist, way. The graphs illustrated in C-F were generated by numerical solution of ordinary differential equations (B) describing the example system shown in A.
